# Supplementary material for: The Evolution and Characterization of the RNA Interference Pathways in Lophotrochozoa
Source: Genome Biol Evol. 2024 May 7;16(5):evae098. doi: 10.1093/gbe/evae098 (PMC11114477; doi:10.1093/gbe/evae098)
Supplement: evae098_Supplementary_Data [file evae098_supplementary_data.zip › Supplementary_materials.zip!/selected_miRNAs/NoGe_selected_miRNAs/novel_miRNA_families_pdf_files/NMRB01000152.1_Notospermus_geniculatus_strain_Ushimado_scaffold152,_whole_genome_shotgun_sequence_5838.pdf]

Provisional ID	:	NMRB01000152.1_Notospermus_geniculatus_strain_Ushimado_scaffold152,_whole_genome_shotgun_sequence_5838
Score total	:	23319.1
Score for star read(s)	:	3.9
Score for read counts	:	23310.4
Score for mfe	:	1.8
Score for randfold	:	0
Score for cons. seed	:	3
Total read count	:	45734
Mature read count	:	45719
Loop read count	:	0
Star read count	:	15

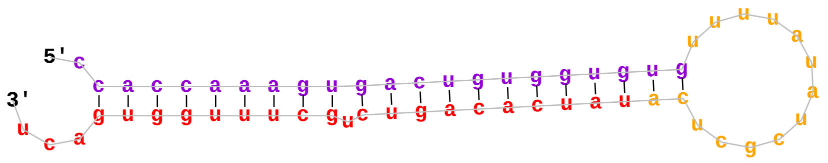

Genome coordinates: NMRB01000152.1 414145-414204

pre-miRNA sequence:
CCACCAAAGTGACTGTGGTGTGCCACCAAAGTGACTGTGGTGTGTTTTATATCGCTCATATCACAGTCTGCTTTGGTGACT

Mature sequence: TATCACAGTCTGCTTTGGTGACT

BLAST results for the pre-miRNA sequence against the *Lineus longissimus* genome assembly:

Scaffold	Start	Stop	Identity	e-value
OU343002.1 1	11593246	11593187	86.885	2.20e-10

```
Nge_novel_miRNA        CCACCAAAGTGACTGTGGTGTGTT--TTATATCGCTCATATCACAGTCTGCTTTGGTGACT
Llo_genome             CCACCAAAGTGATTGTGATGTGTTGGTTGTA-AACTCATATCACAGTCTGCTTTGGTGACT
```
